# Refractory human cytomegalovirus infection without evidence of genetic resistance in the UL-54 and UL-97 genes in a pediatric hematopoietic stem cell transplant recipient: a case report

**DOI:** 10.3389/fmed.2024.1335969

**Published:** 2024-02-02

**Authors:** Alejandra Pando-Caciano, Ketty Adid Escudero-Ramirez, Jackeline Carol Rodríguez-Torres, Holger Maita-Malpartida

**Affiliations:** ^1^Department of Cellular and Molecular Sciences, School of Science and Philosophy, Universidad Peruana Cayetano Heredia, Lima, Peru; ^2^Sub Unidad Integral Especializada del Paciente de Progenitores Hematopoyéticos, Instituto Nacional de Salud del Niño San Borja, Lima, Peru; ^3^Sub Unidad de Investigación e Innovación Tecnológica, Instituto Nacional de Salud del Niño San Borja, Lima, Peru

**Keywords:** HSCT, cytomegalovirus infection, antiviral resistance, ganciclovir, foscarnet, cidofovir, case report

## Abstract

Cytomegalovirus (CMV) infection is a common complication in patients undergoing hematopoietic stem cell transplantation (HSCT). Management of refractory CMV infections, especially in developing countries, can be challenging due to the limited availability of second and third-line antiviral drugs or alternative treatments. Here, we present a case of an 8 years-old patient diagnosed with acute myeloid leukemia. Eight months post-diagnosis, the patient underwent TCR-αβ^+^/CD19^+^-depleted haploidentical HSCT. Both the donor and recipient tested positive for anti-CMV IgG and negative for IgM antibodies. Before transplantation, the patient received CMV prophylaxis in the form of intravenous ganciclovir. Post-transplantation, the patient exhibited oscillating CMV viral loads and was diagnosed with a refractory infection. Treatment with ganciclovir, foscarnet, and cidofovir was unsuccessful. Sequencing of UL-54 and UL-97 genes was performed to rule out potential resistance to first-line treatment. Ten months after the HSCT, the child died from hypovolemic shock due to gastrointestinal bleeding. This is the first case reported in Peru and Latin America of a refractory CMV infection in a pediatric HSCT recipient without evidence of clinical symptoms and CMV genetic resistance. This case demonstrates the need for alternative treatments to manage refractory CMV infections, especially in haploidentical HSCT cases where drug resistance is frequent (~15%). Furthermore, this case highlights the importance of using highly sensitive genetic tools to detect mutations associated with virus resistance in a broader range of the viral genome.

## Introduction

Human herpesvirus 5 or human cytomegalovirus (CMV) is a member of the Herpesviridae family (1). CMV seroprevalence in Latin America (60%–90%) is significantly higher than in Europe or North America (2). The infection among immunocompetent individuals is usually asymptomatic, but among transplanted individuals, CMV can cause fatal diseases such as pneumonia, enteritis, cystitis, and encephalitis (3). Additionally, infection can cause graft failure due to the inhibition of the myelopoiesis (4).

Post-transplant CMV infection monitoring relies on weekly qPCR-based detection and quantification of blood viral load (5). This approach enables preemptive antiviral therapy to prevent end-stage organ disease (5). Approved antiviral agents for CMV infections include ganciclovir, valganciclovir, foscarnet, cidofovir, letermovir, and maribavir (6, 7).

One main challenge in managing CMV infection in transplant recipients is refractory infections, which may be related to genetic or non-genetic mechanisms (8). The significant incidence of resistant CMV, especially among haploidentical HSCT recipients (~15%) (9), makes the need for quick and accurate detection of resistant cases evident. Typically, the choice of therapeutic approaches is adjusted according to the specific resistance profile of the virus with the aim of achieving early remission of the infection. CMV drug resistance is evaluated by conventional PCR and sequencing of the UL-54 (phosphotransferase) and UL-97 (DNA polymerase) genes (10).

We report here the case of a high-risk pediatric patient with acute myeloid leukemia who developed a refractory CMV infection. Despite treatment with ganciclovir, valganciclovir, foscarnet, and cidofovir, the infection persisted. This is the first case reported in Peru and Latin America of refractory CMV infection in a pediatric HSCT recipient without evidence of clinical symptoms or genetic resistance.

This case highlights the need for highly sensitive genetic tests to characterize mutations associated with CMV resistance to drugs and for alternative treatments to manage refractory CMV infections in order to extend the survival chances of individuals undergoing HSCT.

## Case report

We present the case of an 8 years-old male patient who underwent haploidentical hematopoietic stem cell transplantation (HSCT) for acute myeloid leukemia. The procedure was carried out on November 18th, 2019, at the Instituto Nacional de Salud del Niño San Borja in Lima, Peru. The patient received an allograft from his mother with a 3/6 HLA match after depleting CD19^+^ and TCRαβ^+^ cells.

The patient received chemotherapy according to the ALL-BFM 2009 protocol and achieved complete remission after four blocks of consolidation (11). The conditioning regimen involved the administration of fludarabine, antithymocyte globulin, cyclophosphamide, and total body irradiation. No prophylaxis for graft-versus-host disease (GvHD) was provided before transplantation. The histocompatibility study showed haploidentical compatibility with his mother at the HLA-B and HLA-DQ loci, resulting in the selection of the mother as the transplant donor.

The patient had no active CMV infection prior to transplantation. However, both the recipient and donor tested positive for anti-CMV IgG. Anti-CMV prophylaxis was started 16 days prior to transplantation, with the initial administration of ganciclovir at a dose of 5 mg/kg/12 h for 6 days, suspension of treatment for 7 days, and restart of ganciclovir treatment at a dose of 5 mg/kg/12 h for 3 days.

No immunosuppressive therapy was administered following transplantation. At day +14, immune reconstitution was assessed by absolute neutrophil count, which was greater than 0.5 × 10^9^ cells/L (neutrophil count = 0.6 × 10^9^ cells/L). Recovery of CD8^+^, CD19^+^, and NK cells was faster than CD4^+^ cells. CD4^+^ recovery occurred at 6 months post-HSCT, while CD8^+^, CD19^+^, and NK recovery occurred during the first trimester (Table 1). At day +35, the patient presented complete chimerism with >95% donor cells for all three cell lineages (T, B, and myeloid cells), confirming engraftment without GvHD.

**Table 1:** Immune recovery after HSCT.

Cell lineage	Cell count (cells/μL)			Reference values (cells/μL)
	2 months post-HSCT	3 months post-HSCT	6 months post-HSCT	
CD4^+^	59	286	455	300–2,000
CD8^+^	196	466	541	300–1,800
CD19^+^	655	1,102	1,123	200–1,600
NK	1,225	892	725	90–900

**Figure 1:**
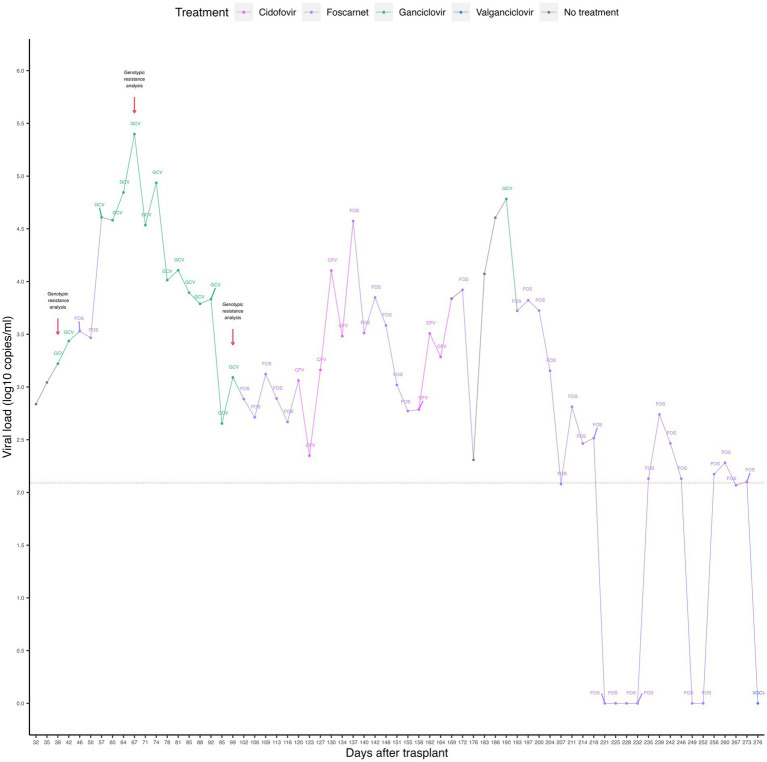
Timeline representing CMV viral loads and antiviral treatments of the case. The dashed red horizontal line represents the limit of detection of the qPCR assay. The red arrows indicate the time points of genotypic resistance analysis. GCV, ganciclovir; FOS, foscarnet; CFV, cidofovir; VGCV, valganciclovir.

The viral load of CMV was quantified by real-time PCR (targeting the 4 IE antigen gene) in peripheral blood (PB) samples. Reactivation of CMV infection was observed on day +32, with the presence of 688 DNA copies/mL. The kinetics of the viral load showed a pattern consistent with a CMV infection refractory to the administered drugs, observing an increase in viral load, accompanied by a fall and a new increase, throughout the post-HSCT period (see Figure 1).

Because of infection reactivation, preemptive treatment was started with intravenous ganciclovir (5 mg/kg/12 h) for 7 days, followed by foscarnet (180 mg/kg/day) for 10 days, ganciclovir (5 mg/kg/12 h) for 46 days, foscarnet (180 mg/kg/day) for 15 days, and cidofovir (5 mg/kg/week) for 4 weeks. The highest viral load (2.5 × 10^5^ copies/mL) was recorded on day +67 during the application of the antiviral treatment and gradually decreased until it reached 223 copies/mL on day +123. An invasive pulmonary infection with small pseudo-nodules in the right lung was also recorded. No causative agents were identified, and the infection was managed with amphotericin B and voriconazole.

On day +127, an increase in viral load from 223 copies/mL to 1,452 copies/mL was observed, restarting treatment with foscarnet (180 mg/kg/day) for 18 days, cidofovir (5 mg/kg/week) for 2 weeks, ganciclovir (5 mg/kg/12 h) for 3 days and foscarnet (180 mg/kg/day) for 80 more days, until day +273. On the fourteenth day after the administration of foscarnet (day +207), the viral load fell below the detection limit (equivalent to 120 copies/mL). However, 4 days later (day +211), an increase in the viral load to 649 copies/ml was observed during the application of foscarnet. The viral load then became negative at day +221, remaining undetectable until day +232. Three days later, virus reactivation was observed with a viral load of 135 copies/mL (day +235), which increased to 549 copies/mL, then decreased to 292 copies/mL, 135 copies/mL, and finally became negative on day +249. The last reactivation was observed on day +256, with a viral load of 149 copies/mL. On day +275, the patient started treatment with oral valganciclovir (15–18 mg/kg/day) for 9 days. After the last reactivation, the viral load became undetectable on day +276, coinciding with the administration of valganciclovir.

Due to our suspicion of a refractory CMV infection, PB samples were analyzed by Sanger sequencing to identify mutations, deletions, insertions, or substitutions in the UL-54 and UL-97 genes known to confer genetic resistance to drugs. Nevertheless, genotypic analyses performed on days +39, +67, and +99 failed to detect modifications compatible with genetic drug resistance.

During the post-HSCT period, the patient showed coinfection with adenovirus and BK polyomavirus, detected by quantitative PCR in plasma and serum. These infections were detected on days +11 and +18 and successfully cleared at days +148 and +188, respectively.

On day +245 of HSCT, the patient was diagnosed with disease relapse by flow cytometry and PCR of bone marrow samples (7.49% of pathological myeloid blasts and 11.14% of AML1-ETO^+^ cells). The biopsy of the pterygoid mass confirmed infiltration of blasts in the right sphenoid maxillary sinus, which extended from the right pterygoid to invade the intracranial extra-axial space at the level of the temporal fossa and right choana. Intravenous cytarabine (30 mg every 24 h) was started for 8 days, and intrathecal cytarabine (30 mg every 24 h) for 3 days, with no effect on disease remission. Chimerism tests revealed mixed chimerism greater than 94%, with donor T, B, and myeloid cell populations greater than 95%, 94%, and 95%, respectively. The patient presented volume growth of the right side of the face, palpebral ptosis, and visual loss of the right eye due to infiltration of blasts. On day +276, the patient was discharged with parental consent and received palliative therapy for pain management with morphine administration.

On day +290, the patient was admitted to the emergency department due to hematemesis followed by fainting. Laboratory tests revealed severe anemia, acute kidney disease due to tumor lysis, leukocytosis with blastemia, and thrombocytopenia. The patient was discharged 2 days later with a poor short-term prognosis. On day +308, the patient was readmitted to the emergency department due to hematemesis. On physical examination, he presented poor general condition, with paleness, tachycardia, hypotension, fever, sunken eyes, multiple ecchymoses, increased volume of the left arm, and chronic malnutrition. Laboratory studies revealed leukocytosis due to the presence of blasts, thrombocytopenia, anemia, high levels of C-reactive protein (354.6 mg/L), urea (80.2 mg/dL), urea nitrogen (37.45 mg/dL), and procalcitonin (86.17 ng/mL). One day after readmission, the patient died of hypovolemic shock due to intestinal bleeding.

## Discussion

This is the first case reported in Peru and Latin America describing persistent CMV infection in a pediatric haploidentical HSCT recipient. CMV infection significantly increases morbidity in transplant patients and represents a major risk factor for survival (12). The incidence of CMV infection in individuals undergoing haploidentical HSCT is significantly higher than in those with matched sibling donors (MSD) or matched unrelated donors (MUD) (85.7%, 39.0%, 55.6%, respectively; *p* < 0.001) (13). Among haploidentical HSCT patients, CMV reactivation occurs in 55% of cases, leading to CMV disease in 5% (14). Receiving a haploidentical HSCT is considered a risk factor for CMV infection development (OR = 4.24, *p* = 0.007) (15).

Refractory infections in HSCT recipients are usually common (12%) (12). Refractory CMV infection is characterized by a viremia increase of more than 1-log_10_ after at least 2 weeks of antiviral treatment (8). It is important to note that some drugs used to treat the CMV infection in the present case could not be administered on time due to shortage at the institution.

The presence of refractory infection constitutes a significant risk for the survival of infected patients (3 years event-free survival: 53% in cases of resistant CMV vs. 87% in cases of susceptible CMV; *p* < 0.001) (12). In the present case, a persistent viral load was observed during the 9 months post-HSCT, with an increase of 2.5-log_10_ compared to the initial viral load of 688 copies/mL. The highest viral load was 2.5 × 10^5^ copies/mL on day +67. These findings confirmed the presence of a refractory CMV infection, which is characterized by fluctuating viral loads.

Initial treatment for CMV infection among HSCT recipients consists of ganciclovir or valganciclovir for at least 2 weeks (3). In case of adverse events (neutropenia or thrombocytopenia) or intolerance, using foscarnet for 2–3 weeks or cidofovir for 2 weeks is recommended (3). Foscarnet administration as a first-line treatment has been suggested for adult patients presenting bone marrow function (16). In cases of mutations conferring high resistance to foscarnet and cidofovir, such as the C592G mutation in the UL97 gene, a switch to ganciclovir is recommended, expecting virus clearance within 14 days (16, 17).

In addition to conventional pharmacological approaches, CMV-specific T-cell therapy (CMV-TCT) has been suggested as a promising treatment option for CMV infections following HSCT. The therapy is suggested for cases of refractory CMV with progressive viral disease such as pneumonitis and colitis (18). Donor-derived CMV-TCT has been proven to be effective in treating CMV infections in pediatric patients undergoing HSCT, with an overall response rate of 89.5% (19). Combining CMV-TCT with foscarnet has demonstrated efficacy in resolving CMV-associated retinitis in a pediatric recipient of haploidentical TCRαβ^+^/CD19^+^ cell-depleted HSCT (20). Notably, this therapeutic strategy has not been reported for HSCT recipients in Peru. Challenges related to logistics and the cost of the T-cell separation process may pose significant obstacles to the future implementation of this therapy in developing countries.

Previous reports suggest combining viral load monitoring with genetic screening to detect mutations in the UL-97 and UL-54 genes for treatment adjustment based on the specific viral resistance profile (21). However, in the presented case, genetic resistance testing during the first 3 months post-HSCT did not reveal any known mutations associated with drug resistance. Contrarily, the highest viral load observed during the entire post-transplant period occurred at day +67 (2.5 × 105 copies/ml), approximately 2 months after the transplant.

Known drug resistance in CMV results primarily from mutations in the UL-97 and UL-54 genes. UL-97 mutations affect ganciclovir activation but do not impact susceptibility to foscarnet or cidofovir (22). UL-54 mutations confer resistance to all available drugs, and their combination with UL-97 mutations results in high-level resistance to multiple drugs (23). Additionally, in transplant recipients, rare mutations associated with letermovir resistance have also been reported in the UL-56 gene, which encodes a component of the viral terminase complex responsible for the cleavage and packaging of viral genome into nascent viral capsids (24–26). Among solid organ transplant recipients, mutations in the UL-97 and UL-54 genes account for 26% of drug resistance cases, whereas mutations in UL-56 account for only 3% of cases (27).

The case presented here is exceptional since there was no response to treatment with ganciclovir, foscarnet, and cidofovir despite the absence of mutations associated with CMV resistance. The last measurement of viral load before the patient’s discharge at day +276, suggested virus clearance by foscarnet, although no additional tests were performed to confirm a possible new reactivation. A similar case of an older adult with chronic myelomonocytic leukemia who underwent HSCT with an MUD donor was previously reported in 2022. The patient presented four episodes of CMV infection with no mutations on the UL-97 and UL-54 genes. Intriguingly, however, analysis of the UL-56 gene revealed an unknown mutation (R246C). This mutation was associated with a superior replicative capacity but did not necessarily lead to a reported increase drug resistance (27). The combination of enhanced replicative capacity and the absence of mutations in UL-97 and UL-54 genes may explain persistent viral loads in cases like the one presented here, suggesting the importance of including the analysis of the UL-56 gene in addition to UL-97 and UL-54 genes in similar cases.

On day +308, the patient died of gastrointestinal bleeding, possibly caused by CMV colitis, similar to a previous report (28). Since gastrointestinal biopsies were not performed, the diagnosis of a probable CMV infection of the gastrointestinal tract could not be verified. Upper and lower gastrointestinal tract infections are more frequent in transplant recipients with underlying hematological conditions than in transplant patients with solid tumors (*p* = 0.02) (29). Therefore, it is essential that healthcare institutions managing transplanted patients have access to tests permitting CMV detection and quantification in the gastrointestinal tract (30). In the case reported here, despite the availability of these laboratory tests, signs of gastrointestinal involvement in the patient did not manifest themselves in the final month before death. During this period, no further testing was performed to confirm probable CMV gastrointestinal disease, as the patient was in palliative care.

In transplant patients, the initial episode of DNAemia is usually asymptomatic (>80%). However, approximately 15% of these individuals have CMV disease when the first viremia is detected, which includes CMV syndrome, pneumonia, and gastrointestinal disease (31). Gastrointestinal bleeding in haploidentical HSCT recipients may occur from day +1 to +892 post-transplantation, with clinical presentations that include rectal bleeding (69%), hematemesis (20%) and melena (9%) (32). Timely treatment is crucial to increase the chances of survival in transplant patients, especially in HSCT patients, as mortality from CMV-induced gastrointestinal can be as high as 42% (29). Hence, monitoring the potential development of CMV disease in transplant patients with persistent viral loads is of critical importance.

Documented cases of HSCT recipients with refractory or resistant CMV infections are scarce worldwide and more typically involve adult solid organ transplants (26, 33–35). In Latin America, only one case of a heart transplant recipient with suspected genetic resistance has been reported, involving a 12 years-old pediatric patient initially treated with ganciclovir upon diagnosis of CMV-DNAemia. As viral loads persisted, foscarnet was administered, resulting in remission of the infection (36). Genetic resistance tests were not conducted, presumably due to the absence of this type of testing in the healthcare institution.

The case reported here highlights the importance of actively monitoring the development of CMV disease in patients with asymptomatic CMV DNAemia and considering alternative therapeutic options. It also highlights the need for more sensitive genetic tests, such as next-generation sequencing (NGS), since standard sequencing fails to detect relevant mutations in 9% of cases (37). The need for this approach is exemplified by the case of an 8 months-old who received an HSTC from an MSD. NSG detected the D588N mutation in the UL-54 gene, while standard sequencing failed to detect it. This mutation is clinically relevant due to its association with resistance to foscarnet, ganciclovir, and cidofovir (38).

In summary, this report described the case of a patient diagnosed with acute myeloid leukemia who underwent haploidentical HSCT and subsequently developed CMV infection refractory to first, second, and third-line treatment. The infection had an atypical asymptomatic presentation with no evidence of genetic resistance in the UL-97 and UL-54 genes. Although thrombocytopenia may have caused gastrointestinal bleeding, it cannot be ruled out that CMV colitis caused this episode.

## Data availability statement

The original contributions presented in the study are included in the article/supplementary material, further inquiries can be directed to the corresponding author.

## Ethics statement

The study was approved by the Comité Institucional de Ética en Investigación, Instituto Nacional de Salud del Niño San Borja. Written informed consent was obtained from the minor(s)’ legal guardian/next of kin for the publication of any potentially identifiable images or data included in this article.

## Author contributions

AP-C: Conceptualization, Formal analysis, Methodology, Writing – original draft. KE-R: Investigation, Writing – original draft. JR-T: Supervision, Writing – review & editing. HM-M: Supervision, Writing – review & editing, Conceptualization, Funding acquisition.
