# Corrigendum: Refractory human cytomegalovirus infection without evidence of genetic resistance in the UL-54 and UL-97 genes in a pediatric hematopoietic stem cell transplant recipient: a case report

**DOI:** 10.3389/fmed.2025.1526535

**Published:** 2025-01-28

**Authors:** Alejandra Pando-Caciano, Ketty Adid Escudero-Ramirez, Jackeline Carol Rodríguez-Torres, Holger Maita-Malpartida

**Affiliations:** ^1^Department of Cellular and Molecular Sciences, School of Science and Philosophy, Universidad Peruana Cayetano Heredia, Lima, Peru; ^2^Sub Unidad Integral Especializada del Paciente de Progenitores Hematopoyéticos, Instituto Nacional de Salud del Niño San Borja, Lima, Peru; ^3^Sub Unidad de Investigación e Innovación Tecnológica, Instituto Nacional de Salud del Niño San Borja, Lima, Peru

**Keywords:** HSCT, cytomegalovirus infection, antiviral resistance, ganciclovir, foscarnet, cidofovir, case report

In the published article, an author name was incorrectly written as [Jackeline Carol Torres-Rodríguez]. The correct spelling is [Jackeline Carol Rodríguez-Torres].

The authors apologize for this error and state that this does not change the scientific conclusions of the article in any way. The original article has been updated.

